# Aminoglycoside induces RIPOR2 translocation and phosphatidylserine externalization via distinct mechanisms

**DOI:** 10.3389/fncel.2025.1636500

**Published:** 2025-08-06

**Authors:** Jinan Li, Michelle Yang, Bo Zhao

**Affiliations:** ^1^Department of Otolaryngology-Head and Neck Surgery, Indiana University School of Medicine, Indianapolis, IN, United States; ^2^Department of Biochemistry and Molecular Biology, Indiana University School of Medicine, Indianapolis, IN, United States; ^3^Stark Neurosciences Research Institute, Indiana University School of Medicine, Indianapolis, IN, United States

**Keywords:** aminoglycoside, RIPOR2, phosphatidylserine, hair cell, ototoxicity, mechanotransduction

## Abstract

Aminoglycosides are widely used to treat severe infections. However, systemically administered AGs preferentially kill cochlear hair cells, resulting in irreversible hearing loss. Recently, we found that AGs induce a rapid translocation of RIPOR2 in hair cells, a process that relies on functional mechanotransduction, subsequently dysregulates the autophagy/mitophagy pathway, and ultimately leads to irreversible hair cell death. Recent studies found that AGs also trigger rapid phosphatidylserine (PS) externalization in hair cells, probably by activating the scramblase activity of TMC1/2, which are the pore-forming subunits of the mechanotransduction channel. To determine whether AG-triggered rapid RIPOR2 translocation and PS externalization are independent, RIPOR2 translocation and PS externalization were extensively investigated in wild-type hair cells treated with AG for different amounts of time. Next, the potential effect of PS externalization on RIPOR2 translocation in hair cells was studied. Finally, we investigated the extent to which cisplatin, a chemotherapy drug that shares several pathological features of ototoxicity with AGs, affects PS externalization and RIPOR2 localization in hair cells. Our results suggest that AG triggers RIPOR2 translocation and PS externalization by independent mechanisms, and that cisplatin and AGs induce hair cell death via distinct molecular pathways.

## Introduction

Hearing loss is one of the most prevalent diseases in the current human population, affecting approximately 1.6 billion people worldwide. The majority of hearing loss cases are linked to exposure to ototoxic aminoglycoside antibiotics (AGs) and the chemotherapy drug cisplatin, which are estimated to cause approximately 19.6 million and 441,000 new cases of hearing loss annually, respectively (Prasad et al., 2024).

Aminoglycosides, including gentamicin, amikacin and streptomycin, are commonly used as a first-line antibiotic therapy due to their effectiveness against gram negative bacteria, their affordability, and their accessibility. AGs act by entering bacteria through hydrophilic porin protein channels (Garneau-Tsodikova and Labby, 2016). After entry, AGs carry out their antibiotic mode of action by binding to the 30 s subunit of the bacterial 70S ribosome, inhibiting the RNA translation initiation complex, and ultimately halting protein synthesis (Krause et al., 2016). This modus operandi contributes to the potent bactericidal nature of this antibiotic class. However, AGs are also known for a strong effect of ototoxicity, leading to hearing loss in 20%–47% patients (Fausti et al., 1992; Huth et al., 2015; Huth et al., 2011). As a result, they are contraindicated in many cases, such as in pregnant patients due to potential risks for congenital hearing loss in the fetus. Despite these concerns, AGs remain a first line treatment for many severe infections, and their frequent use leads to approximately 20 million new cases of hearing loss annually (Prasad et al., 2024). Given the bactericidal efficacy and widespread use of this antibiotic class, the mechanisms that underlie the ototoxic effects of AGs have become a fervent area of study.

In cochlear hair cells, which are specialized mechanosensory cells in the inner ear that convert sound vibrations into electrochemical signals, AGs not only enter the cells primarily through mechanotransduction (MET) channels located near the tips of stereocilia on the apical surface (Alharazneh et al., 2011; Beurg et al., 2009; Kawashima et al., 2011; Li et al., 2022b; Marcotti et al., 2005; Vu et al., 2013), but they also block these MET channels (Marcotti et al., 2005). Notably, these MET channels act like one-way valves for AGs, allowing entry but limiting their exit (Marcotti et al., 2005). As a result, AGs accumulate within the cytoplasm of hair cells, and their increased concentrations initiate a cascade of cellular responses that ultimately lead to hair cell death. Because mammalian cochlear hair cells do not regenerate, the consequences of their death are permanent and result in irreversible hearing loss.

In our previous studies, we found that AGs rapidly trigger the translocation of RIPOR2 from the base of stereocilia to the pericuticular area in hair cells upon entering through the MET channel. Once translocated, RIPOR2 dysregulates the autophagy pathway by interacting with and recruiting GABARAP along with several other autophagy-related proteins (Li et al., 2022b; Li et al., 2023; Li et al., 2025). Notably, inhibiting the expression of several key proteins in the RIPOR2-GABARAP pathway completely prevents AG-induced hair cell death and subsequent hearing loss, which suggests the essential role of this pathway in AG-induced hair cell death (Li et al., 2022b,^2023^; Li et al., 2025).

Notably, Goodyear et al. (2008) reported that AGs induce a rapid externalization of phosphatidylserine (PS) in cochlear hair cells. PS is an aminophospholipid that is preferentially distributed in the inner leaflet of the cell membrane. Its externalization is usually facilitated by lipid scramblases, which are responsible for the ATP-independent translocation of lipids between lipid bilayers (Sakuragi and Nagata, 2023). Unlike in many other cell types, where PS externalization typically serves as an early marker of apoptosis, its externalization in AG-exposed hair cells appears to be reversible and may not directly result in hair cell apoptosis (Goodyear et al., 2008). Remarkably, TMC1 and TMC2, the pore-forming subunits of the MET channel (Holt et al., 2024; Kawashima et al., 2011; Pan et al., 2018; Pan et al., 2013), exhibit structural similarities to TMEM16, a family of lipid scramblases and ion channels (Ballesteros et al., 2018; Kalienkova et al., 2021; Pan et al., 2018). The inhibition of the MET channel by MET inhibitors such as benzamil triggers rapid PS externalization, probably through the activation of the scramblase activity of TMCs (Ballesteros and Swartz, 2022; Beurg et al., 2025; Peineau et al., 2025). Therefore, AG-induced PS externalization is likely mediated through MET channel inhibition, similar to the effects of several other MET blockers (Ballesteros and Swartz, 2022). However, given that functional MET is also required for AG uptake and the subsequent translocation of RIPOR2, it remains unclear whether AG-induced rapid PS externalization and RIPOR2 translocation are mechanistically linked or occur through independent pathways.

In addition to its critical role in AG-induced hair cell death (Li et al., 2022b), RIPOR2 is also required for the proper morphogenesis of stereocilia (Diaz-Horta et al., 2018; Zhao et al., 2016). Genetic deletion of RIPOR2 results in disorganized stereocilia, highlighting the importance of this protein in hair cell morphogenesis (Diaz-Horta et al., 2018; Zhao et al., 2016). Similarly, hair cells lacking several other proteins that colocalize with RIPOR2 at the base of stereocilia, such as GRXCR2 and taperin, also exhibit stereocilia disorganization, suggesting a coordinated role of these proteins in shaping stereocilia architecture (Avenarius et al., 2018; Liu et al., 2018). However, whether some of these base-localized proteins, such as GRXCR2 and taperin, regulate AG-induced RIPOR2 translocation remains unclear.

In this study, we systematically investigated RIPOR2 translocation and PS externalization. Our findings suggest that AG-triggered RIPOR2 translocation and PS externalization exhibit distinct dynamic properties and function independently of each other. Our findings also suggest that several other proteins localized at the stereocilia base, including GRXCR2 and taperin, are not required for AG-induced RIPOR2 translocation. Finally, we found that cisplatin, another ototoxic drug, does not induce RIPOR2 translocation or PS externalization, despite its ototoxicity also requiring functional MET (Kitcher et al., 2019; Li et al., 2022a; Maruyama et al., 2024; Thomas et al., 2013), suggesting that cisplatin induces hair cell death through a mechanism distinct from that of AG.

## Results

### AG triggers mechanotransduction-dependent RIPOR2 translocation and PS externalization in cochlear hair cells

**FIGURE 1:**
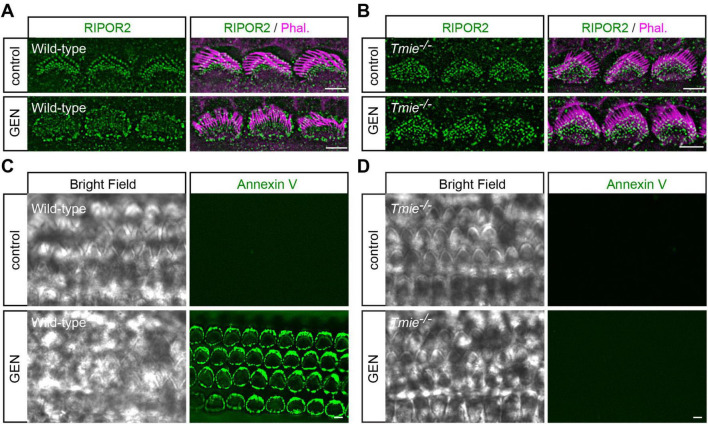
AG triggers mechanotransduction-dependent RIPOR2 translocation and PS externalization in cochlear hair cells. **(A,B)** Wild-type **(A)** and *Tmie*^–/–^ **(B)** P4 cochlear explants were treated with 1 mM AG antibiotic gentamicin for 15 min, then fixed and stained for RIPOR2 (green) and phalloidin (magenta) to visualize stereocilia. Notably, in wild-type hair cells, RIPOR2 translocated robustly from the stereocilia base to the pericuticular area, a phenomenon not observed in *Tmie*^–/–^ hair cells. **(C,D)** Wild-type **(C)** and *Tmie*^–/–^ **(D)** P4 cochlear explants were treated with 1 mM GEN for 15 min in medium containing fluorescent Annexin V. In wild-type hair cells, robust phosphatidylserine (PS) externalization was evident as fluorescent Annexin V signals, whereas *Tmie*^–/–^ hair cells did not exhibit this response. Scale bars: 5 μm.

Consistent with previous findings (Li et al., 2022b), treating cochlear explants dissected from postnatal day 3 to 5 (P3-P5) wild-type C57/BL6 mice with 1 mM gentamicin (GEN), a representative AG antibiotic, for 15 min resulted in a rapid RIPOR2 translocation in hair cells, as revealed by immunostaining (Figure 1A). The GEN treatment also resulted in PS externalization, as detected by fluorescent dye-conjugated Annexin V in wild-type hair cells (Figure 1C). Notably, functional MET is required for both phenomena, as neither RIPOR2 translocation nor PS externalization was observed in hair cells lacking TMIE (Figures 1B, D), an essential subunit of the MET channel in auditory hair cells (Cunningham et al., 2020; Zhao et al., 2014).

### Distinct dynamic properties of RIPOR2 cycling and PS externalization in response to AG treatment

**FIGURE 2:**
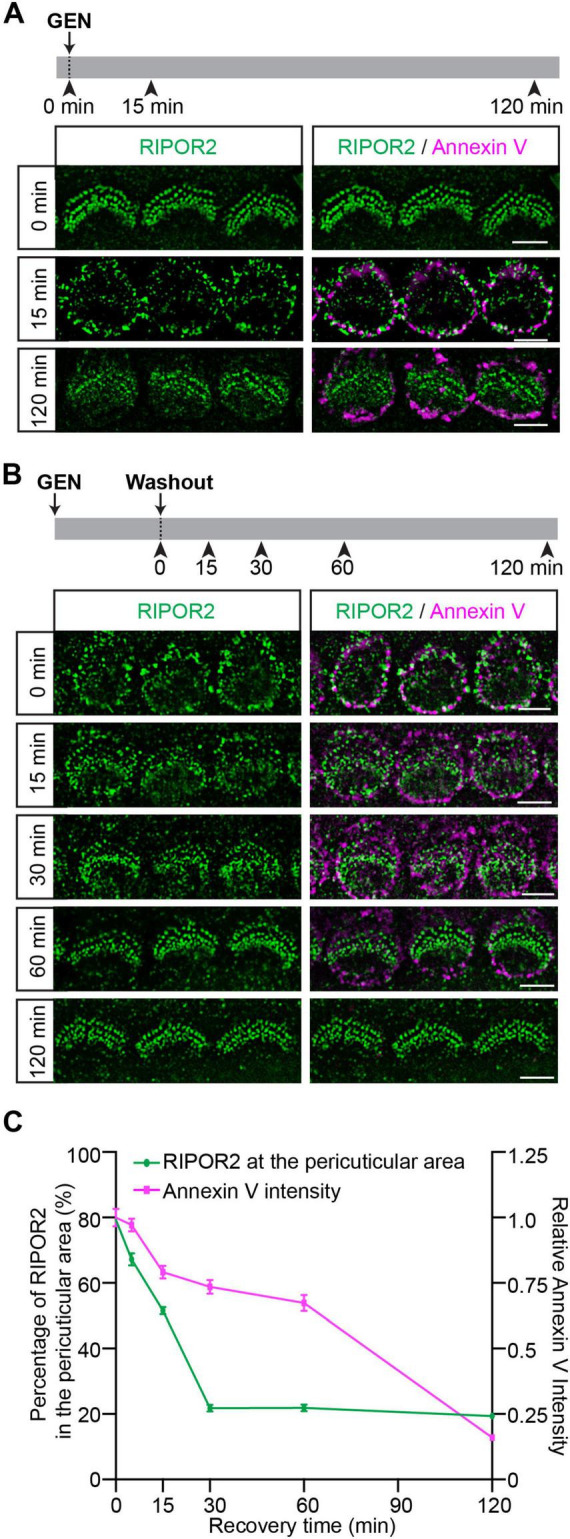
Distinct dynamic properties of RIPOR2 cycling and PS externalization in response to AG treatment. **(A)** Wild-type cochleae were treated with GEN in a medium containing fluorescent Annexin V. Note, RIPOR2 was not colocalized with Annexin V. **(B)** Wild-type cochleae were treated with 1 mM GEN for 15 min in the medium containing Annexin V, washed and then incubated in medium without GEN and Annexin V for various amounts of time. RIPOR2 rapidly cycled back, having mostly returned to stereocilia base after 30 min, while the Annexin V signal needed ∼2 h to disappear. **(C)** Percentage of RIPOR2 in the pericuticular area in comparison to the Annexin V signal. The fluorescence intensity of Annexin V was normalized against the intensity of Annexin V before GEN washout. Data are represented as the mean ± SEM (100 IHCs from at least three mice were measured in each group). Scale bars: 5 μm.

To extensively investigate RIPOR2 translocation and PS externalization, wild-type hair cells were treated with 1 mM GEN for either 15 min or 2 h in a medium containing fluorescent dye-conjugated Annexin V (Figure 2A). Incubation was followed by fixation for immunostaining. Consistent with previously published data (Ballesteros and Swartz, 2022; Beurg et al., 2025; Goodyear et al., 2008; Li et al., 2022b; Peineau et al., 2025), untreated cells showed no Annexin V signal, and RIPOR2 localized predominantly at the base of their stereocilia. After 15 min of GEN exposure, Annexin V signals appeared on the apical surface of hair cells, while RIPOR2 translocated to the pericuticular area (Figure 2A). Notably, no colocalization of RIPOR2 and Annexin V was detected. After 2 h of GEN treatment, the majority of RIPOR2 returned to the base of the stereocilia, whereas Annexin V signals remained largely unchanged (Figure 2A). This result suggests that RIPOR2 translocation and PS externalization exhibit distinct dynamics.

To further characterize their differences, we employed an alternative treatment strategy previously described by Goodyear et al. (2008). Cochlear explants were treated with GEN for 15 min, washed, and then incubated in GEN-free medium for various amounts of time (Figure 2B). Thirty minutes after GEN removal, most RIPOR2 had relocalized to the stereociliary base, while Annexin V signals persisted at the apical surface (Figures 2B, C). Consistent with previous findings (Goodyear et al., 2008), Annexin V signals gradually diminished and was nearly disappeared 2 h post-washout (Figures 2B, C). Notably, Annexin V intensity was shown to fall much later than RIPOR2 concentration at the pericuticular region, with the decline in intensity becoming steeper at 60 min after GEN washout, while the RIPOR2 percuticular concentration initially exhibits a steep decline before plateauing at 30 min (Figures 2B, C). These differential recovery kinetics suggest that GEN-induced RIPOR2 translocation and PS externalization are regulated by distinct mechanisms.

### Benzamil induces PS externalization without triggering RIPOR2 translocation

**FIGURE 3:**
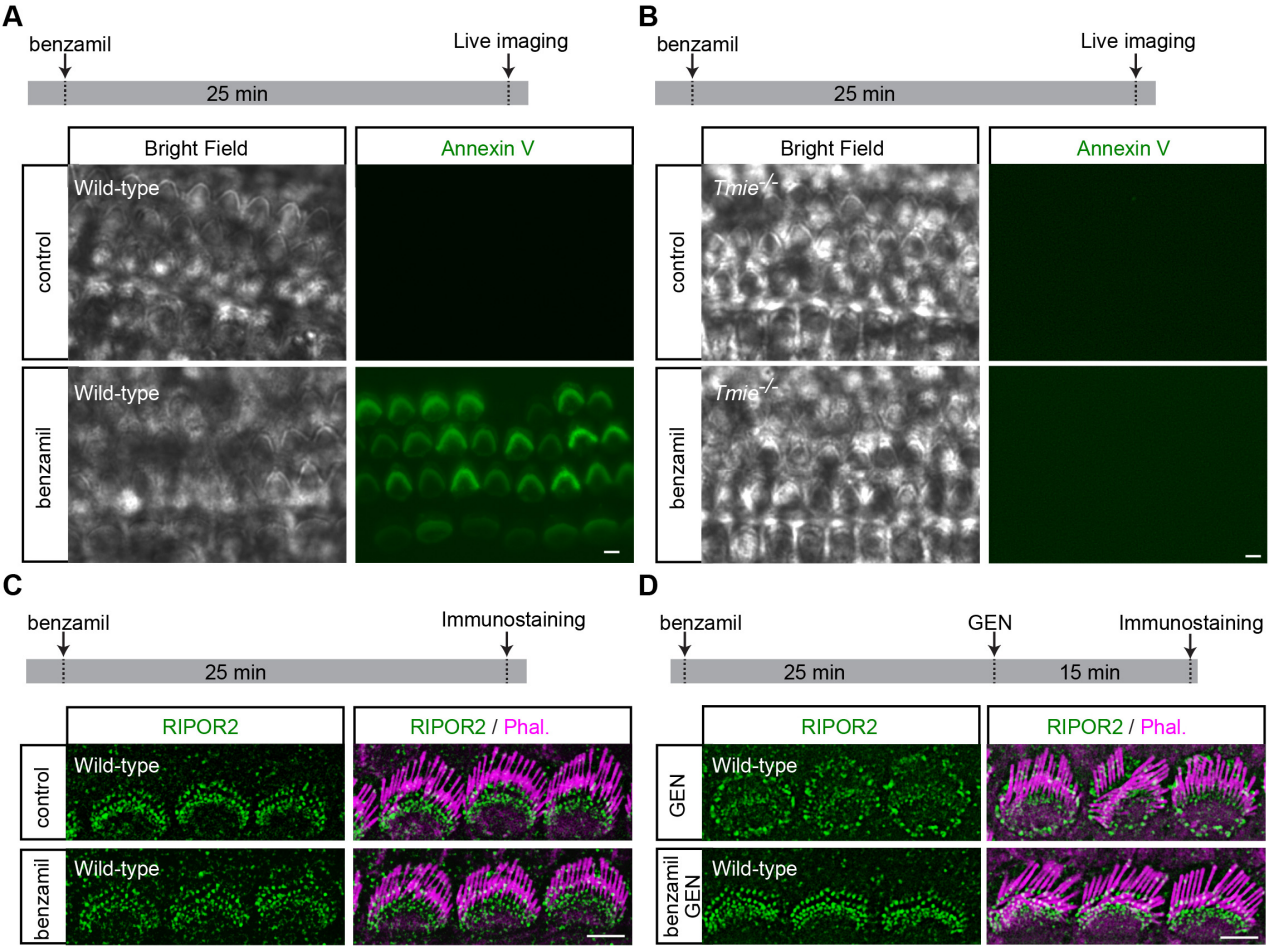
Benzamil induces PS externalization without triggering RIPOR2 translocation. **(A,B)** Wild-type **(A)** and *Tmie*^/^ **(B)** P4 cochlear explants were treated with 0.1 mM benzamil for 25 min in the medium containing fluorescent Annexin V. The robust PS externalization indicated by fluorescent Annexin V signals in wild-type hair cells but not in *Tmie*^/^ hair cells. **(C)** Wild-type P4 cochlear explants were treated with 0.1 mM benzamil for 25 min and then fixed for immunostaining. No RIPOR2 translocation was observed after benzamil treatment. **(D)** Wild-type P4 cochlear explants were treated with 1 mM GEN for 15 min, either following a 25-min pretreatment with 0.1 mM benzamil or without pretreatment, and were then fixed for immunostaining. Benzamil inhibited GEN-induced RIPOR2 translocation. Scale bars: 5 μm.

Next, we sought to determine whether PS externalization alone is sufficient to induce RIPOR2 translocation. Previous studies have shown that multiple MET channel blockers, such as benzamil, can trigger PS externalization in hair cells (Ballesteros and Swartz, 2022; Beurg et al., 2025; Peineau et al., 2025). Following established protocols (Ballesteros and Swartz, 2022; Beurg et al., 2025; Peineau et al., 2025), we treated wild-type cochlear explants with 0.1 mM benzamil for 25 min. As expected, benzamil induced robust PS externalization in wild-type hair cells (Figure 3A). Consistent with previous findings (Peineau et al., 2025), *Tmie*-deficient hair cells, which lack MET current (Zhao et al., 2014), do not exhibit PS externalization upon benzamil treatment (Figure 3B), suggesting that the MET channel is indeed essential for PS externalization. Remarkably, further immunostaining revealed that benzamil treatment does not induce RIPOR2 translocation in wild-type hair cells (Figure 3C). Instead, pretreating wild-type hair cells with benzamil prevented GEN-induced RIPOR2 translocation, probably by inhibiting GEN uptake via MET channel blockade (Figure 3D). These findings suggest that PS externalization alone is insufficient to trigger RIPOR2 translocation.

### GRXCR2 or taperin is not required for AG-triggered RIPOR2 translocation

The loss of RIPOR2 or several other proteins that colocalize with RIPOR2 at the stereocilia base, such as GRXCR2 and taperin, results in morphological defects of stereocilia, suggesting a coordinated function of these proteins in maintaining stereocilia architecture (Avenarius et al., 2018; Diaz-Horta et al., 2018; Liu et al., 2018; Zhao et al., 2016). However, it remains unclear whether any of these base-localized proteins influence AG-induced RIPOR2 translocation. Thus, we sought to determine the extent to which GRXCR2 and taperin is essential for AG-induced RIPOR2 translocation.

**FIGURE 4:**
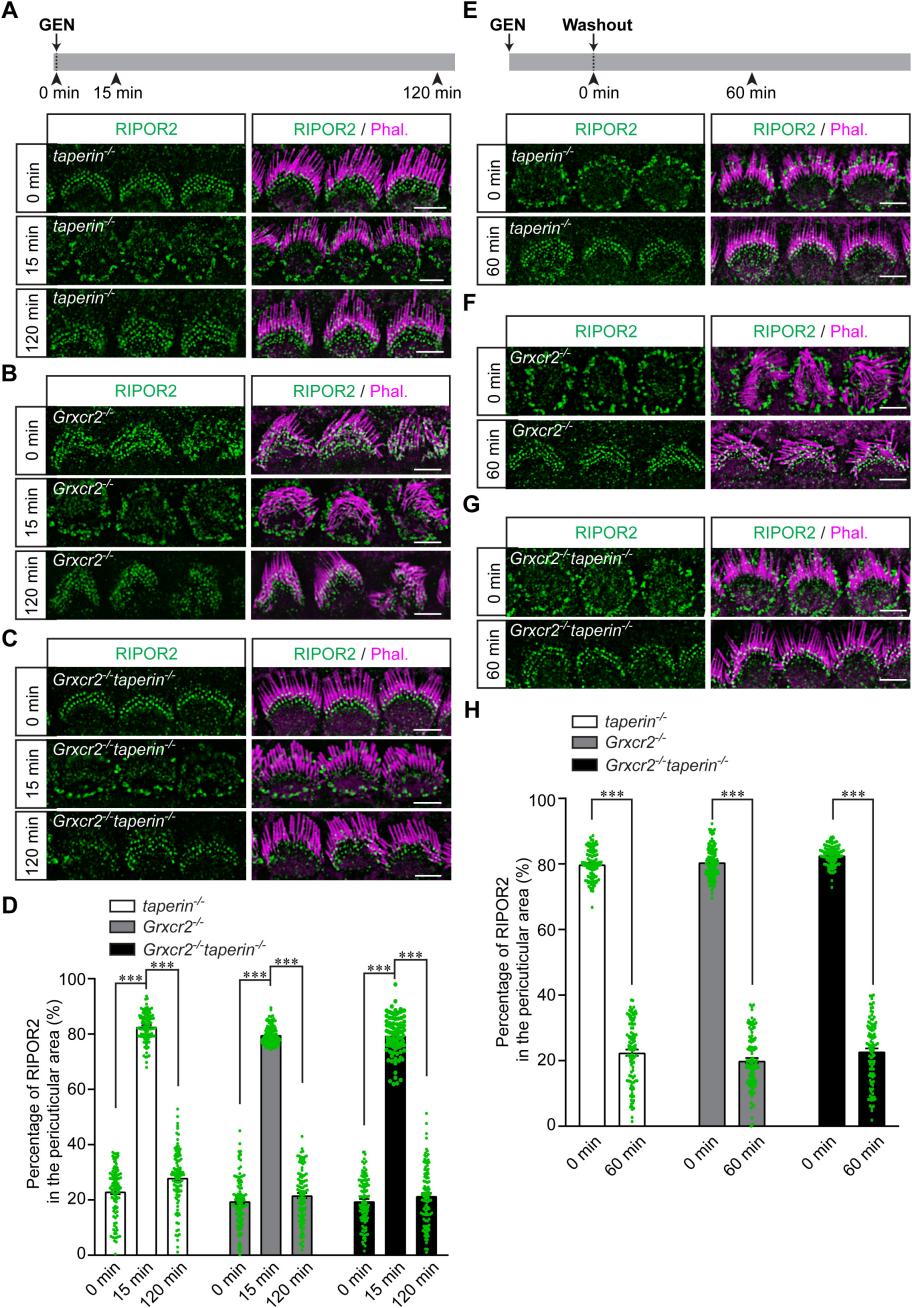
Translocation of RIPOR2 in *taperin*- and *Grxcr2*-deficient hair cells. **(A–C)** Cochleae from P4 *taperin*^–/–^ **(A)**, *Grxcr2*^–/–^ **(B)** or *Grxcr2*^–/–^ taperin^–/–^ **(C)** mice were dissected and treated with 1 mM GEN for 15 min or 2 h. The rapid translocation of RIPOR2 was triggered by GEN in these mutant hair cells. **(D)** Quantification of the percentage of RIPOR2 located in the pericuticular area in IHCs as shown in panels **(A–C)**. Data are represented as the mean ± SEM (100 IHCs from at least three mice were measured in each group). Asterisks indicate statistically significant differences from the group treated with GEN for 15 min, compared with either the untreated group or the group treated with GEN for 2 h, within each genotype (***p < 0.001 by unpaired two-sided *t*-test). **(E–G)** Cochleae from P4 *taperin*^–/–^ **(E)**, *Grxcr2*^–/–^ **(F)** or *Grxcr2*^–/–^ taperin^–/–^ **(G)** mice were dissected and treated with 1 mM GEN for 15 min. Then GEN was washed out and cochlear explants were incubated in the medium without GEN for 60 min. Samples were fixed and immunostaining was performed. RIPOR2 cycled back to the stereociliary base in *taperin*^–/–^, *Grxcr2*^–/–^ and *Grxcr2*^–/–^ taperin^–/–^ hair cells after 60 min. **(H)** Quantification of the percentage of RIPOR2 located in the pericuticular area as shown in panels **(E–G)**. Data are represented as the mean ± SEM (100 IHCs from at least three mice were measured in each group). Asterisks indicate statistically significant differences from the group treated with GEN for 15 min compared with the group treated with GEN followed by a 2-h washout, within each genotype (***p < 0.001 by unpaired two-sided *t*-test). Scale bars: 5 μm.

Consistent with previous results (Liu et al., 2018), stereocilia were disorganized in cochlear explants dissected from P4 *Grxcr2^–/–^* mice but were only mildly affected in hair cells from *taperin*^–/–^ or *Grxcr2*^–/–^taperin^–/–^ mutant mice (Figures 4A–C). RIPOR2 was localized at the base of the stereocilia in all these types of hair cells, suggesting that neither taperin nor GRXCR2 is required for the localization of RIPOR2 to the stereociliary base (Figures 4A–C). Then, cochlear explants dissected from *Grxcr2*^–/–^, *taperin*^–/–^ or *Grxcr2*^–/–^taperin^–/–^ mice were treated with 1 mM GEN for either 15 min or 2 h. Like what occurred in hair cells dissected from wild-type mice, RIPOR2 rapidly translocated between the stereociliary base and pericuticular area in hair cells dissected from all these mutant mice (Figures 4A–D), suggesting that the translocation of RIPOR2 triggered by AGs does not require GRXCR2, taperin, or the structural integrity of the stereocilia. To investigate whether GRXCR2 and/or taperin is critical for RIPOR2 translocating back to the stereociliary base, *Grxcr2*^–/–^, *taperin*^–/–^ or *Grxcr2*^–/–^taperin^–/–^ hair cells were treated with 1 mM GEN for 15 min. Then, GEN was washed out and hair cells were cultured in a medium without GEN for 60 min (Figures 4E–G). Tissues were then fixed and immunostained. Remarkably, in hair cells of all the aforementioned genotypes, RIPOR2 re-localized to the base of the stereocilia (Figures 4E–H). These findings suggest that GRXCR2 and taperin are non-essential for AG-induced RIPOR2 translocation between the stereociliary base and pericuticular area in hair cells.

### Cisplatin cannot trigger either RIPOR2 translocation or PS externalization

**FIGURE 5:**
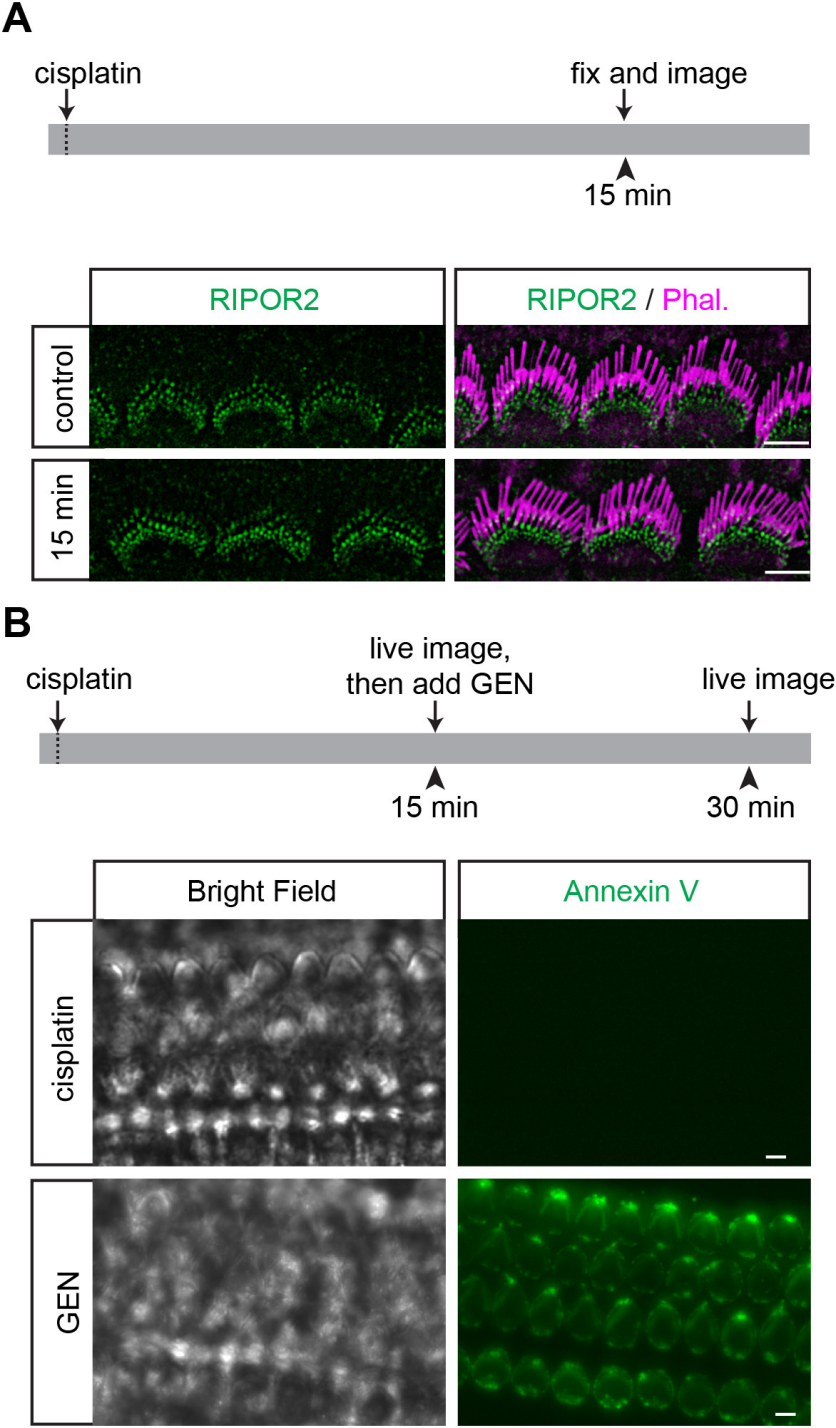
Cisplatin cannot trigger either RIPOR2 translocation or PS externalization. **(A)** Wild-type P4 cochlear explants were treated with 1 mM cisplatin for 15 min and then fixed for immunostaining. No RIPOR2 translocation was observed after cisplatin treatment. **(B)** Wild-type P4 cochlear explants were treated with 0.3 mM cisplatin for 15 min and then treated with 1 mM GEN for 15 min. Cisplatin can neither trigger PS externalization nor prevent GEN-induced PS externalization. Scale bars: 5 μm.

Multiple lines of evidence suggest that cisplatin and AGs share several pathological features in their ototoxic effects (Steyger, 2021). For instance, inhibition of MET can prevent the entry of both AGs and cisplatin into cochlear hair cells, thereby protecting against hair cell death (Kawashima et al., 2011; Kitcher et al., 2019; Li et al., 2022a; Li et al., 2022b; Maruyama et al., 2024; Thomas et al., 2013). Additionally, in chick cochlear hair cells, cisplatin has been shown to inhibit MET currents (Kimitsuki et al., 1993). However, 1 mM cisplatin treatment did not induce RIPOR2 translocation in wild-type hair cells (Figure 5A). To determine whether cisplatin induces PS externalization in murine hair cells, cochlear explants from P4 mice were exposed to 0.3 mM cisplatin for 15 min in the presence of fluorescent dye-conjugated Annexin V. Following cisplatin treatment, no Annexin V signal was detected in the hair cells (upper panels, Figure 5B). Subsequently, the cochlear explant was treated with GEN for 15 min, resulting in a strong Annexin V signal on the apical surface of the hair cells (lower panels, Figure 5B). These findings suggest that cisplatin does not trigger RIPOR2 translocation or activate the scramblase activity of TMC proteins to induce PS externalization, indicating that, although cisplatin and AGs share some cellular-level features in their ototoxic effects, they probably induce hair cell death through distinct molecular mechanisms.

## Discussion

In this study, we found that although AG induces both rapid RIPOR2 translocation and PS externalization, and both processes require functional MET, these two events are probably governed by distinct molecular mechanisms. Several lines of evidence support this conclusion. First, while RIPOR2 returns to the base of the stereocilia during sustained GEN treatment, PS externalization persists after RIPOR2 dissipates from the pericuticular region (Figure 2A). Second, RIPOR2 re-localization and the reversal of PS externalization undergo distinct kinetics from each other following GEN washout (Figures 2B, C). Third, benzamil-triggered PS externalization does not influence RIPOR2 localization at the stereocilia base (Figure 3C). Fourth, pretreatment with benzamil prevents AG-induced RIPOR2 translocation (Figure 3D).

Given the structural similarities between TMC1/2 and TMEM16F, a major lipid scramblase in mammalian cells, it is plausible that TMC1/2 also possess scramblase activity (Ballesteros et al., 2018; Kalienkova et al., 2021; Pan et al., 2018), particularly when their MET function is inhibited (Ballesteros and Swartz, 2022). Supporting this hypothesis, hair cells lacking both TMC1 and TMC2 not only lose MET-evoked currents, but also completely fail to externalize PS in response to MET channel blockers (Ballesteros and Swartz, 2022; Kawashima et al., 2011; Pan et al., 2013; Peineau et al., 2025). Consistently, in *Tmie*^–/–^ hair cells, which lack evocable MET currents (Zhao et al., 2014), PS externalization cannot be triggered by either GEN or benzamil. Interestingly, although TMC2 single gene knockout mice retain some MET function (Kawashima et al., 2011) and show benzamil-induced scramblase activity (Peineau et al., 2025), *Tmie*^–/–^ hair cells do not exhibit PS externalization in response to either GEN or benzamil (Figures 1B, D), despite retaining TMC2 localization in the stereocilia (Cunningham et al., 2020). This suggests that TMIE may be a crucial component for the TMC-mediated scramblase activity required for PS externalization in cochlear hair cells, in addition to its known role as an essential MET channel subunit. Further *in vitro* and/or *ex vivo* studies are needed to test this hypothesis. Additionally, it would be of interest to further explore the mechanisms that regulate the switch between MET channel function and scramblase activity of the MET complex, specifically on how MET inhibitors can simultaneously activate the scramblase function while blocking MET currents, and how membrane homeostasis influences–and/or is influenced by–the MET channel and its scramblase activities.

Given that AG binds to RIPOR2 both *in vitro* and *in vivo* (Li et al., 2022b), it is plausible that this interaction triggers RIPOR2 translocation. The AG antibiotics family is composed of both natural products and semisynthetic derivatives produced by a variety of actinomycetes. Since mammalian cells do not synthesize these antibiotics, it remains unclear whether RIPOR2 translocation can also be triggered by endogenous small molecules under specific physiological or pathophysiological conditions. Notably, many receptors and signaling pathways in mammalian cells respond to both exogenous compounds and endogenous ligands. For example, opioid receptors are activated by plant-derived opioids as well as endogenous peptides such as enkephalins and endorphins, while cannabinoid receptors similarly respond to both external cannabinoids and endogenous endocannabinoids. Likewise, it might be possible that, like exogenous AGs, certain endogenous small molecules, peptides, or proteins may bind to RIPOR2 and trigger its translocation under conditions that have yet to be identified.

While RIPOR2 colocalizes with several proteins at the base of stereocilia and potentially works together with these proteins to regulate stereocilia morphogenesis, at least two of these proteins, GRXCR2 and taperin, are not required for RIPOR2 localization in hair cells and do not affect its AG-induced translocation (Figure 4). In our previous studies, we also found that neither taperin nor GRXCR2 undergoes the same AG-triggered localization change seen with RIPOR2 (Li et al., 2022b). These findings suggest that AG elicits a rapid and specific response in hair cells that is limited to a few proteins such as RIPOR2, rather than a broad or non-specific protein redistribution.

Although PS externalization is frequently considered an early marker of apoptosis in many other cell types, the blockage of the MET with its inhibitors, such as benzamil, induces PS externalization without triggering apoptosis in cochlear hair cells. In fact, blocking MET with benzamil or other inhibitors protects hair cells from AG- and cisplatin-induced cell death (Kitcher et al., 2019; Vlasits et al., 2012). Interestingly, ototoxic cisplatin causes hair cell death without inducing PS externalization. These findings suggest that PS externalization can occur independently of hair cell death, and that cochlear hair cell death may proceed without necessarily initiating PS externalization. This raises an interesting question about the role of PS externalization in other forms of hair cell death. While the essential the role of RIPOR2-mediated autophagy pathway in AG-induced hair cell death has been revealed, the contribution of PS externalization to AG ototoxicity requires further investigation, ideally through the use of a yet-to-be-identified inhibitor that specifically blocks the scramblase activity of the MET complex.

Previous studies found that functional MET is essential for cisplatin uptake and the subsequent hair cell death it causes (Li et al., 2022a; Maruyama et al., 2024; Thomas et al., 2013), which is similar to the phenomena observed with AG. Despite its ability to block MET in chick hair cells (Kimitsuki et al., 1993), cisplatin fails to induce PS externalization in murine hair cells under the conditions tested (Figure 5), unlike several other MET inhibitors (Ballesteros and Swartz, 2022). Moreover, cisplatin does not prevent GEN-induced PS externalization, which depends on functional MET. These findings suggest that cisplatin may inhibit MET through a mechanism distinct from AG, provided it can inhibit MET in murine hair cells. Additionally, the inability of cisplatin to induce RIPOR2 translocation implies that it activates an intracellular signaling pathway that is different from the one triggered by AG once inside hair cells. To develop effective therapeutic strategies for preventing cisplatin-induced hair cell death and the resulting permanent hearing loss, it is crucial to further investigate the precise mechanisms by which cisplatin blocks MET, enters hair cells, and then activates cytotoxic signaling cascades.

## Materials and methods

### Animals

Mice were maintained on a 12 h day/night cycle with adequate food and water under specific pathogen-free conditions. *Tmie*^–/–^ (MGI:5784557), *Grxcr2*^–/–^ (MGI:6281113), *taperin*^–/–^ (MGI:6281115), and *Grxcr2*^–/–^taperin^–/–^ mice have been described previously (Liu et al., 2018; Zhao et al., 2014). The primers used to genotype *Tmie*^–/–^ mice were: 5′- GGCTCGGTATCTACAGCGAAAGGCGGCC -3′ and 5′- TGCCT GGCTCTGACTAGTTTCTGCAC -3′. Primers used to genotype *Grxcr2*^–/–^ mice were: 5′- TCTTCCTACAGTGGCCGAGT -3′ and 5′- TGAATGTGAGCGAGATACCG -3′. Primers used to genotype *taperin*^–/–^ were: 5′- CTGGAAACGGGAGATCCTTG -3′ and 5′- GAAGCCTGGCGCTGACTC -3′. All animal experiments were carried out in accordance with the National Institutes of Health Guide and were approved by the Institutional Animal Care and Use Committee of Indiana University School of Medicine. More than three mice from at least two litters per group were used in each experiment.

### Cochlear explant culture and immunostaining

Cochlear explant culture and immunostaining were performed as previously described (Li et al., 2022b). Briefly, cochleae from P3-P5 pups were dissected and cultured in DMEM/F12 medium (Life technologies) at 37°C in a 5% CO2 humidified incubator for 4–12 h. Cochlear explants were then treated with GEN (cat# G1264, MilliporeSigma), benzamil (cat# 33-801-0, Fisher Scientific) or cisplatin (cat# 232120, MilliporeSigma). Samples were fixed in 4% paraformaldehyde (PFA) in Hank’s Balanced Salt Solution (HBSS) for 20 min, followed by removal of the tectorial membrane after washing in HBSS. Tissues were blocked for 20 min at room temperature (RT) in HBSS containing 5% bovine serum albumin (BSA) and 0.5% Triton X-100, then incubated overnight at 4°C with primary antibodies diluted in HBSS with 1% BSA. After washing in HBSS, samples were incubated with secondary antibodies diluted in HBSS with 1% BSA for 2 h at RT and mounted using Prolong^®^ Antifade Reagents (cat# P36971, Life technologies). Image stacks were acquired from the middle regions of cochleae using a Leica DM6 FS automated deconvolution microscope with a 100× objective (HCX PL APO 100×/1.40–0.70 OIL). Antibodies against RIPOR2 have been generated and described previously (Li et al., 2022b). Additional reagents included: Alexa Fluor 488-phalloidin (cat# A12379, Life technologies), Alexa Fluor 568-phalloidin (cat# A12380, Life technologies), Alexa Fluor 488 goat anti-rabbit (cat# A-11070, Life technologies), Alexa Fluor 546 goat anti-rabbit (cat# A-11071, Life technologies). ImageJ (NIH) was used to measure the fluorescence intensity of RIPOR2.

### Annexin V labeling and live cell imaging

Annexin V labeling and live cell imaging was performed according to the published literatures (Ballesteros and Swartz, 2022; Beurg et al., 2025; Li et al., 2022b; Peineau et al., 2025). In brief, cochleae from postnatal pups were dissected and cultured in DMEM/F12 medium (cat# 21041025, Life technologies) at 37°C in a 5% CO_2_ humidified incubator for 4–12 h. Cochlear explants were then labeled using Alexa Fluor 488 Annexin V (cat# A13201, Life technologies) or Alexa Fluor 568 Annexin V (cat# A13202, Life technologies) immediately following the addition of GEN, benzamil, or cisplatin to the culture medium. Stacked Images were then captured from the middle regions of cochleae by a DM6 FS automated deconvolution microscope (Leica) using a 63× objective (HCX APO L63×/0.90 water immersion).

### GEN, benzamil and cisplatin treatment

Cochleae dissected from postnatal pups were cultured in DMEM/F12 medium (Life technologies) at 37°C in a 5% CO_2_ humidified incubator for 4–12 h. The cochleae were treated with 1 mM GEN (cat# G1264, MilliporeSigma), 0.1 mM benzamil (cat# 33-801-0, Fisher Scientific), or either 0.3 mM or 1 mM cisplatin (cat# 232120, MilliporeSigma), either in the absence of Annexin V followed by fixation for immunostaining, or in the presence of fluorescent Annexin V followed by live cell imaging. Stacked images were collected from the middle region of the cochlea by a DM6 FS automated deconvolution microscope (Leica) using either a 100× objective (HCX PL APO 100×/1.40–0.70 OIL) or a 63× objective (HCX APO L63×/0.90 water immersion).

### Quantification and statistical analysis

Details on precise numbers, sample sizes, number of repetitions, and statistical tests are provided in the figure legends. Data are presented as mean ± standard error of the mean (SEM). Statistical significance was determined using a two-tailed unpaired Student’s *t*-test (*p < 0.05, **p < 0.01, ***p < 0.001).

## Data Availability

The original contributions presented in this study are included in this article/supplementary material, further inquiries can be directed to the corresponding author.
